# Model-Based Methods to Translate Adolescent Medicine Trials Network for HIV/AIDS Interventions Findings Into Policy Recommendations: Rationale and Protocol for a Modeling Core (ATN 161)

**DOI:** 10.2196/resprot.9898

**Published:** 2019-04-16

**Authors:** Anne M Neilan, Kunjal Patel, Allison L Agwu, Ingrid V Bassett, K Rivet Amico, Catherine M Crespi, Aditya H Gaur, Keith J Horvath, Kimberly A Powers, H Jonathon Rendina, Lisa B Hightow-Weidman, Xiaoming Li, Sylvie Naar, Sharon Nachman, Jeffrey T Parsons, Kit N Simpson, Bonita F Stanton, Kenneth A Freedberg, Audrey C Bangs, Michael G Hudgens, Andrea L Ciaranello

**Affiliations:** 1 Division of General Academic Pediatrics Massachusetts General Hospital Boston, MA United States; 2 Medical Practice Evaluation Center Massachusetts General Hospital Boston, MA United States; 3 Department of Epidemiology and Center for Biostatistics in AIDS Research Harvard T.H. Chan School of Public Health Boston, MA United States; 4 Departments of Pediatric and Adult Infectious Diseases Johns Hopkins University School of Medicine Baltimore, MD United States; 5 Division of Infectious Diseases Department of Medicine Massachusetts General Hospital Boston, MA United States; 6 University of Michigan School of Public Health Ann Arbor, MI United States; 7 Department of Biostatistics Fielding School of Public Health University of California Los Angeles Los Angeles, CA United States; 8 St. Jude's Children's Research Hospital Memphis, TN United States; 9 Division of Epidemiology and Community Health School of Public Health University of Minnesota Minneapolis, MN United States; 10 Department of Epidemiology Gillings School of Global Public Health University of North Carolina at Chapel Hill Chapel Hill, NC United States; 11 Hunter College of the City University of New York New York, NY United States; 12 Institute for Global Health & Infectious Diseases University of North Carolina at Chapel Hill Chapel Hill, NC United States; 13 Arnold School of Public Health University of South Carolina Columbia, SC United States; 14 Center for Translational Behavioral Research Florida State University Tallahassee, FL United States; 15 State University of New York Stony Brook, NY United States; 16 Medical University of South Carolina Charleston, SC United States; 17 Hackensack Meridian School of Medicine at Seton Hall University Nutley, NJ United States; 18 Division of General Internal Medicine Massachusetts General Hospital Boston, MA United States; 19 Department of Health Policy and Management Harvard T.H. Chan School of Public Health Boston, MA United States; 20 Department of Biostatistics Gillings School of Global Public Health University of North Carolina at Chapel Hill Chapel Hill, NC United States

**Keywords:** adolescent, costs and cost analysis, health policy, HIV, medication adherence, modeling, retention in care, youth

## Abstract

**Background:**

The United States Centers for Disease Control and Prevention estimates that approximately 60,000 US youth are living with HIV. US youth living with HIV (YLWH) have poorer outcomes compared with adults, including lower rates of diagnosis, engagement, retention, and virologic suppression. With Adolescent Medicine Trials Network for HIV/AIDS Interventions (ATN) support, new trials of youth-centered interventions to improve retention in care and medication adherence among YLWH are underway.

**Objective:**

This study aimed to use a computer simulation model, the Cost-Effectiveness of Preventing AIDS Complications (CEPAC)-Adolescent Model, to evaluate selected ongoing and forthcoming ATN interventions to improve viral load suppression among YLWH and to define the benchmarks for uptake, effectiveness, durability of effect, and cost that will make these interventions clinically beneficial and cost-effective.

**Methods:**

This protocol, ATN 161, establishes the ATN Modeling Core. The Modeling Core leverages extensive data—already collected by successfully completed National Institutes of Health–supported studies—to develop novel approaches for modeling critical components of HIV disease and care in YLWH. As new data emerge from ongoing ATN trials during the award period about the effectiveness of novel interventions, the CEPAC-Adolescent simulation model will serve as a flexible tool to project their long-term clinical impact and cost-effectiveness. The Modeling Core will derive model input parameters and create a model structure that reflects key aspects of HIV acquisition, progression, and treatment in YLWH. The ATN Modeling Core Steering Committee, with guidance from ATN leadership and scientific experts, will select and prioritize specific model-based analyses as well as provide feedback on derivation of model input parameters and model assumptions. Project-specific teams will help frame research questions for model-based analyses as well as provide feedback regarding project-specific inputs, results, sensitivity analyses, and policy conclusions.

**Results:**

This project was funded as of September 2017.

**Conclusions:**

The ATN Modeling Core will provide critical information to guide the scale-up of ATN interventions and the translation of ATN data into policy recommendations for YLWH in the United States.

## Introduction

### Background

Approximately 60,000 youth are living with HIV in the United States. Youth living with HIV (YLWH) have poorer outcomes than adults living with HIV, including lower rates of diagnosis, engagement, retention, and virologic suppression [1,2]. Established in 2001 by the Maternal and Pediatric Infectious Disease Branch of the Eunice Kennedy Shriver National Institutes of Child Health and Development, the Adolescent Medicine Trials Network for HIV/AIDS Interventions (ATN) has conducted rigorous evaluations of interventions to improve medication adherence, retention in care, and viral load (VL) suppression among YLWH [3]. ATN is the only national clinical research network that specifically studies adolescents aged 12 to 24 years living with HIV and at risk for acquiring HIV. ATN collaborations have included the United States Centers for Disease Control and Prevention; the Health Resources and Services Administration; the AIDS Clinical Trials Group; the HIV Vaccine Trials Network; the HIV Prevention Trials Network; the International Maternal, Pediatric, and Adolescent AIDS Clinical Trial (IMPAACT) Network; and the Microbicide Trials Network. This ATN began with new National Institutes of Health (NIH) support in 2016 to fund youth-focused projects that aim to reduce risk factors for adolescents at risk of acquiring HIV and to promote behaviors related to adherence and engagement with care for those living with HIV. ATN currently supports 22 protocols [3].

By projecting outcomes beyond the time horizon of traditional studies and thereby permitting estimates of long-term clinical outcomes and cost-effectiveness, computer-based health policy models can add substantial value to clinical trials and observational studies [4]. Projecting such long-term estimates is particularly important for studies among YLWH, for whom the health effects of poor virologic control may not manifest for years or decades [5]. Models can also combine data from multiple sources and compare a wide range of possible interventions, leveraging the extensive data collected within ATN and other studies into timely guideline and policy recommendations [6]. The Cost-effectiveness of Preventing AIDS Complications (CEPAC)-computer simulation models [7] of HIV infection in infants, children, and adults have been used to inform health policy related to HIV prevention [8,9], testing [10-12], and care [13-18], both in the United States and internationally. CEPAC model-based work has been cited in national HIV care guidelines for the United States, Brazil, Chile, Mexico, France, and Colombia, among others, as well as in the World Health Organization (WHO) guidelines [19-23]. For example, a CEPAC-Pediatrics model-based analysis projected that use of lopinavir/ritonavir in children younger than 3 years as first-line antiretroviral therapy (ART) led to longer life-expectancy and was cost-saving compared with first-line use of nevirapine; this analysis helped inform the WHO’s recommendation in 2013 of a lopinavir/ritonavir-based regimen for first-line ART in that age group [16,24]. To date, few HIV modeling or cost-effectiveness studies have been conducted among youth; most have focused on HIV screening and prevention [25-32]. Previous work has not incorporated age- and time-varying changes in adolescent and young adult health-related behavior among YLWH.

**Table 1:** National Institutes of Health–supported studies from the Adolescent Medicine Trials Network for HIV/AIDS Interventions and the International Maternal, Pediatric, and Adolescent AIDS Clinical Trials Network included in the proposed analysis.

Study; time^a,b^	Title	Years	Age at enrollment	N (13-24)^c^	Population^d^
ATN^e^ 061 [33-36]; 2.9 years	T-cells in ART^f^ deintensification	2007-2010	18-24 years	130 (all)	NPHIVY^g^
ATN 106/086 [37-40]; 1 year	Health status and behavioral risk factors	2011-2012	12-24 years	2196 (all)	NPHIVY and PHIVY^h^
ATN 125 [41,42]; 1.5 years	Treatment at ATN sites	2015-2017	13-24 years	922 (all)	NPHIVY
P^i^1055 [41,43,44]; 1.8 years	Psychiatric conditions in PHIVY	2005-2006	6-17 years	294 (199)	PHIVY
P1066 [45-48]; 1 year	RAL^j^ safety, PK^k^, effectiveness	2007-2013	1 month to 19 years	126 (71)	PHIVY
P1074 [49-51]; 5.3 years	Long-term outcomes	2009-2014	0-24 years	1236 (all)	NPHIVY and PHIVY
P1093 [52,53]; 2 years	Dolutegravir-based ART	2011-2018	1 month to 18 years	160 (23)	PHIVY

^a^Mean or median follow-up time.

^b^Minimum key data for all studies: viral loads, cluster of differentiation 4 (CD4) cell count, ART regimens, opportunistic infections, sexually transmitted infections, pregnancy, and other clinical diagnoses.

^c^Total N (n aged 13-24 years): 4904 (4777).

^d^Population: primarily NPHIVY or PHIVY.

^e^ATN: Adolescent Medicine Trials Network for HIV/AIDS Interventions.

^f^ART: antiretroviral therapy.

^g^NPHIVY: nonperinatally HIV-infected youth.

^h^PHIVY: perinatally HIV-infected youth.

^i^P: pediatric.

^j^RAL: raltegravir.

^k^PK: pharmacokinetic.

### Objectives

This protocol will leverage existing data from successfully completed NIH-supported studies (Table 1) to inform the development of novel approaches for modeling critical components of HIV disease and care in YLWH. As ATN investigators study new interventions to improve VL suppression among YLWH, the CEPAC-Adolescent computer simulation model will be developed to define the benchmarks for uptake, effectiveness, durability of effect, and cost that will make these interventions clinically beneficial and cost-effective. In addition, as new data emerge from ongoing ATN trials about the effectiveness of these interventions, the computer simulation model will serve as a flexible tool to project the long-term clinical impact and cost-effectiveness of these interventions. This project will, therefore, provide critical information to guide the scale-up of ATN interventions and the translation of ATN data into policy recommendations for YLWH in the United States.

## Methods

### Adolescent Medicine Trials Network for HIV/AIDS Interventions Structure and Establishment of the Modeling Core

**Figure 1:**
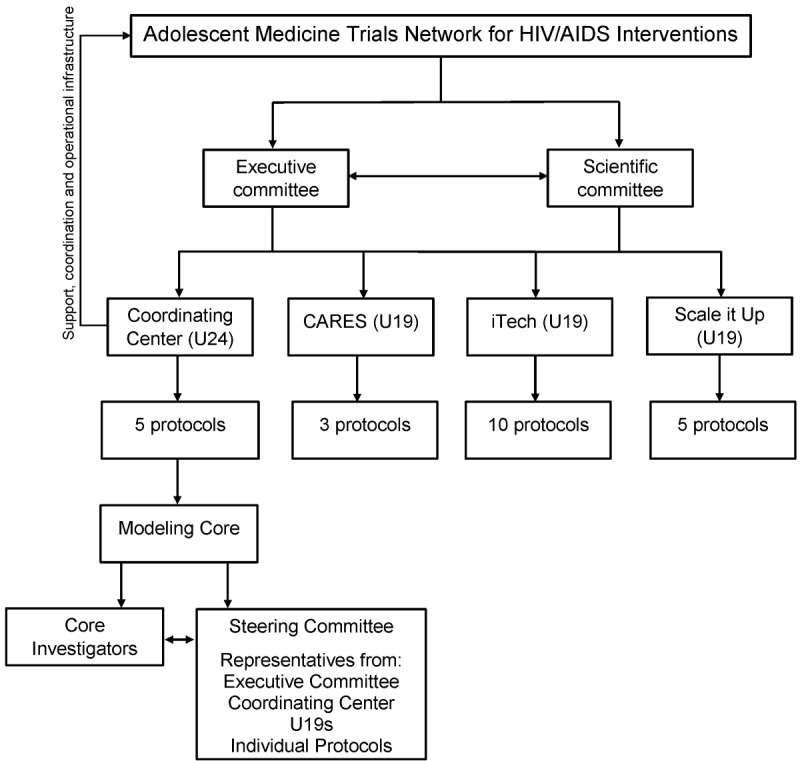
Organizational structure of the Adolescent Medicine Trials Network for HIV/AIDS Interventions (ATN).

The ATN structure consists of 3 ATN research program projects (U19s) and a Coordinating Center (U24; Figure 1). Each of the 3 ATN research program projects (U19) has a well-defined research focus supported by core infrastructures as well as participant recruitment and enrollment capacity. These research program projects are as follows:

- Comprehensive Adolescent Research and Engagement Studies [54], a comprehensive community-based project that aims to optimize the HIV prevention and treatment continuum for at-risk and acutely infected youth as well as youth with established HIV infection.
- iTech [55], a research program that aims to impact the HIV epidemic by conducting innovative, interdisciplinary research using technology-based interventions across the HIV prevention and care continuum for adolescents and young adults.
- Scale it Up [56], a research program that aims to assess and enhance the real-world effectiveness, implementation, and scalability of theoretically based and developmentally tailored interventions focused on improving HIV treatment and prevention self-management for youth.

Each research program project (U19) supports several individual protocols [54-56]. The ATN Coordinating Center (U24) is located at the University of North Carolina at Chapel Hill. The Coordinating Center provides support, coordination, and operational infrastructure to ATN. The Coordinating Center also supports several stand-alone protocols such as “A Triggered, Escalating, Real-Time Adherence Intervention,” which uses electronic-dose monitoring to inform an adherence intervention for youth without virologic suppression. The Coordinating Center also supports the Modeling Core.

The Modeling Core has established a Modeling Core Steering Committee that will meet regularly and include Modeling Core investigators, at least 1 principal investigator or liaison from each of the 3 ATN research program projects (U19s) and the ATN Coordinating Center, protocol chairs or representatives from stand-alone trials for which modeling is planned, and additional interested ATN investigators.

ATN investigators in the Modeling Core Steering Committee will provide feedback on the derivation of data inputs, design of new model structure within the CEPAC-Adolescent model, and selection of policy analyses to perform. Once specific ATN studies are identified as potential candidates for modeling analyses, the Modeling Core investigators will work with relevant protocol teams to ensure that data likely to be useful for later modeling are collected prospectively in each study.

After the Modeling Core Steering Committee has determined which policy analyses will be performed, project teams will be assembled for each analysis. Each project team will include Modeling Core investigators and the protocol chair or a representative from the trial being analyzed. Project team members have expertise in multiple relevant areas including epidemiology, health services research, economics, intervention science, implementation science, behavioral science, clinical trials development, and the clinical care of YLWH. Project teams will help develop the research question, identify any additional structural simulation model modifications, provide input on needed data parameters (eg, help identify potential issues of population mismatch for parameters derived from different sources), and review preliminary model results (eg, for face validity and identifying key sensitivity analyses). Abstracts, presentations, and manuscripts presenting model results will be reviewed in accordance with the ATN publications policy.

**Categories of key outcomes (specific events within each listed category will also be analyzed separately).**

Categories of key outcomes:

- Centers for Disease Control and Prevention (CDC) HIV clinical diagnoses (CDC-A, B, and C)
- Severe or life-threatening, non-HIV–related diagnoses (eg, pneumococcal events)
- Chronic non-HIV-related diagnoses (eg, cardiac and renal disease and malignancy)
- Medication toxicity (division of AIDS ≥Grade 2)
- Psychiatric events
- Sexually transmitted infections
- Pregnancy or pregnancy outcomes
- Death

### Scientific Objectives’ Overview

#### Objective 1

Objective 1 was to determine rates of key clinical events for YLWH engaged in care stratified by age, CD4 cell count, and antiretroviral (ARV) and VL status in completed and ongoing NIH-supported studies. Using ClinicalTrials.gov [57], studies were reviewed that included YLWH aged 13 to 24 years at the US sites, and collected data related to CD4 cell count, VL, and ART regimens as well as clinical event data during the era of modern ART. Selected studies were conducted within the 2 largest NIH-sponsored national networks supporting clinical trials and observational studies in youth affected by HIV—the ATN and the IMPAACT Network. Incidence rates of opportunistic infections; HIV and non-HIV events (Textbox 1); and mortality based on age, sex, patterns of CD4 count and VL, and ARV use among YLWH will be evaluated in completed and ongoing NIH-sponsored studies (Table 1) in accordance with individual data use agreements.

#### Objective 2

Objective 2 was to develop the CEPAC-Adolescent model—a simulation model to reflect unique characteristics of YLWH.

The CEPAC-Adolescent simulation model will be developed to reflect the unique characteristics of YLWH. The foundational inputs of the expanded model will be populated with estimates from completed and ongoing NIH-supported studies (Table 1) derived in Objective 1 as well as other published sources. As new data emerge from the ATN or other sources related to clinical events, resource utilization, and specific interventions, model inputs will be updated.

#### Objective 3

Objective 3 was to use the simulation model to project the clinical impact, cost, and cost-effectiveness of selected interventions evaluated in ATN.

The Modeling Core Steering Committee will work with the ATN Executive Committee and the ATN External Scientific Panel to prioritize ATN studies for model-based analyses, based on data availability and the most relevant questions in health care policy for YLWH each year. The Modeling Core Steering Committee functions will include activities such as providing feedback on the costing perspectives to be used, the primary outcome to be modeled, secondary outcome measures to be included, and the types of economic estimates to be derived from the model.

### Design for Objective 1

The design for Objective 1 was to determine rates of key clinical events for YLWH engaged in care stratified by age, CD4 cell count, and ARV and VL status in completed and ongoing NIH-supported studies.

Incidence rates of key clinical events (Textbox 1) will be described based on current age, sex, current CD4, current ARV use, and VL as well as mode of HIV acquisition (perinatally HIV-infected youth [PHIVY] or nonperinatally HIV-infected youth [NPHIVY]) [58]. These data will permit assigning risks of clinical events to simulated patients in the simulation model developed in Objective 2.

#### Population and Data Sources

Formal requests were approved to analyze data from 4800 YLWH in completed NIH-supported studies after appropriate data use agreement and network approvals were secured (Table 1). These studies include observational studies, nonrandomized interventions, and a randomized trial. All include youth aged 13 to 24 years at study entry. The primary focus of each study ranged widely, from determining the safety and efficacy of ARV medications to evaluating clinical, immunological, and psychiatric outcomes. All included a minimum set of key outcomes needed for this analysis, and all clinical events were recorded using comparable diagnostic codes. Protocols and data collection forms from all studies will be reviewed to understand how data can be harmonized among studies, as has been done in previous analyses [58]. Resource use input parameters will be derived from adolescent intervention or trial-specific data where available, as in previous work [29,59]. New data emerging from ongoing studies will be integrated into the model.

#### Data Management

Data analysis concept sheets and data use agreements have been approved for these analyses by individual networks as well as through the Eunice Kennedy Shriver National Institute of Child Health and Development Data and Specimen Hub repository [60]. Data will be cleaned (when applicable), harmonized, and safely stored at the Center for Biostatistics in AIDS Research at the Harvard TH Chan School of Public Health, which is compliant with federal regulations governing information security.

#### Outcomes

Clinical events that impact short- and long-term (lifetime) outcomes and health care costs, such as the occurrence of specific opportunistic infections, non-AIDS-defining illnesses, sexually transmitted infections, pregnancy, and psychiatric events, within the categories listed in Textbox 1 will be analyzed.

#### Statistical Analysis

Incidence rates of each outcome will be estimated, stratified by mode of HIV acquisition and the combination of time-varying age (7-12, 13-17, 18-24, and 25-30 years), CD4 cell count (<200, 200-499, and ≥500/µL), and VL and ARV status, as in previous work [58]. The VL or ARV status will be categorized as follows: (1) suppressive ARVs—VL less than 400 copies/mL and any prescribed ARVs, (2) nonsuppressive ARVs—VL 400 copies/mL or more and prescribed ARVs expected to be suppressive, and (3) no ARVs—VL 400 copies/mL or more and no prescribed ARVs [58]. Linear interpolation between CD4 cell counts and log_10_-transformed VL will be used to estimate dates when strata thresholds are crossed. These estimated dates will allow us to determine baseline strata and calculate total person-time contributed to each stratum.

As in previous work, trends in incidence rates of outcomes across ordinal age, CD4 cell count, and VL/ARV categories, stratified by mode of HIV acquisition, will be assessed using Poisson regression models, accounting for within-subject correlation with robust SEs [61]. The hypothesis that higher rates of clinical events will be associated with person-time spent with lower CD4 counts, older age, and at higher VL will be examined [58]. VL of 400 copies/mL or more was selected based on historic lower levels of detection for assays used during the study period [58].

We will also advance approaches to describe and predict the trajectories of CD4, VL, and care engagement over time. Locally weighted smoothing plots will be used to obtain a graphical summary of CD4 and VL trajectories over time by mode of HIV acquisition and baseline age [62]. On the basis of visual inspection, linear regression or piecewise linear regression models will be fitted to obtain slope parameters for CD4 and VL over follow-up time among subjects with at least 2 available measures. Baseline covariates such as mode of HIV acquisition, age, CD4 count, VL, and ART regimen will be added to these regression models to assess association with observed CD4 and VL trajectories. To determine whether there are any important differences between subjects with longitudinal CD4 and VL data and those missing such data, baseline characteristics will be compared between these 2 populations.

If there are sufficient numbers of YLWH who miss visits or are lost to follow-up, these will also be used to identify patterns of care followed by distinct subgroups such as those who are in care, those who are care interrupters, and those who are not in care. Latent trajectory groups will be identified from the study data with group-based trajectory modeling [63,64]. After we identify groups of participants following similar trajectories, in a secondary analysis, we will assess associations between baseline characteristics of study participants and membership in particular trajectory groups. In the CEPAC-Adolescent model, these attributes will be used to account for heterogeneity in care engagement.

### Design for Objective 2

The design for Objective 2 was to develop a simulation model to reflect the unique characteristics of YLWH.

#### Current Model Structure

The CEPAC-Adult and -Pediatric models are Monte Carlo, state-transition models of HIV disease and treatment [7,8,17,18,29,65]. The models simulate people living with HIV with user-specified characteristics including age, CD4 cell count, VL, and treatment history, from model entry until death. In the absence of effective ART, CD4 cell counts decline monthly; with VL suppression, CD4 cell counts rise at user-specified rates. In each month, modeled patients face risks of key clinical events, such as opportunistic infections, other illnesses, and mortality, determined by current age and current CD4 cell count. Patients can also initiate or continue ARVs, with subsequent VL suppression or virologic failure, and can be lost to follow-up. Onward transmission risk is determined by pooled cohort VL levels using rates derived from published estimates (eg, 2.06/100PY transmission with HIV RNA of 3000-10,000 copies/mL) from adolescents, where available [66,67].

**Figure 2:**
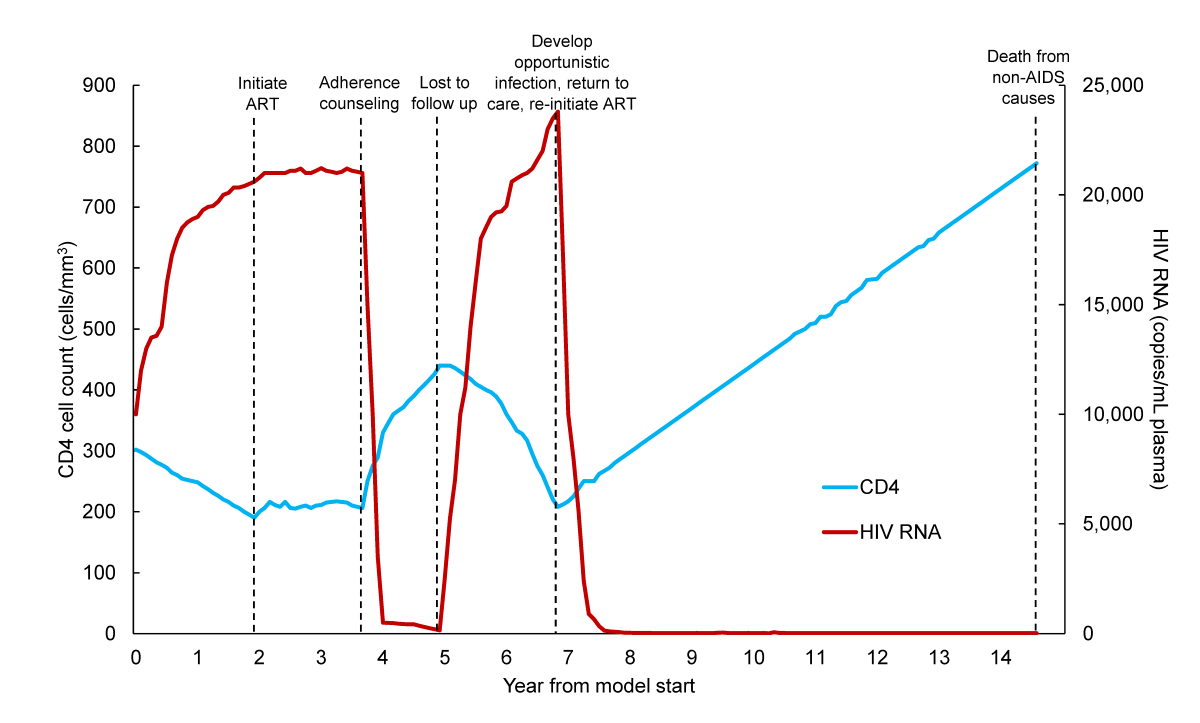
Sample simulated patient trace. CD4 cell count (cells/mm3) is presented on the vertical left-most axis and in the blue line. HIV RNA (copies/mL) is presented on the vertical right-most axis and in the red line. The horizontal axis shows years from model start. The dashed lines mark key clinical events for a simulated patient: initiating ART but failing to suppress HIV RNA and without improvement in CD4 cell count; receiving adherence counseling leading to HIV RNA suppression and improvement in CD4 cell count; becoming lost to follow up with subsequent rise in HIV RNA and decline in CD4 cell count; developing an opportunistic infection resulting in returning to care and reinitiating ART with subsequent HIV RNA suppression and improvement in CD4 cell count; and eventual death from non-AIDS-related causes. ART: antiretroviral therapy.

Patients are simulated within the model one at a time; the model tracks their clinical course, from time of entry into the model until death. Upon a patient’s death, the model records summary statistics, and a new patient then enters the model. This continues until the last patient in a cohort dies and exits the model, at which time the model tallies clinical events, durations spent in each health state, monthly life and quality-adjusted life expectancies, and costs. State transitions are stochastic and determined by the Mersenne Twister random number generator algorithm [68] that was adapted for use in C++, the programming language of CEPAC. When finished running, model output can be extracted and analyzed by the user, as shown in Figure 2, which traces a simulated patient’s CD4 cell count and HIV RNA over the course of several important clinical events.

Adolescent-specific patterns of medication adherence and care engagement will be simulated based on work in Objective 1. Currently, in the CEPAC models, clinical event risk, retention in care, and adherence vary between individual people, but do not vary over time or with changes in development or life events. The new model structure will be developed to reflect age- and time-varying adolescent- and young adult-specific aspects of HIV disease progression and care for YLWH based on these patterns. The model structure will account for heterogeneity—the specific additions to the model structure will be informed by the data generated through activities conducted as a part of Objective 1. Additional details of the existing CEPAC models, including flowcharts, a user guide, and sample patient traces can be found on the CEPAC website [7].

#### Translating Objective 1 Data Into Model Inputs

Incidence rates of clinical events (Textbox 1, Objective 1) will be converted into monthly event probabilities. During each patient-month for which a specific set of characteristics apply (age, CD4, and ARV/VL category), these data will be used to assign a modeled risk of each key clinical event over the next 30-day period.

Resource utilization and cost data related to HIV care, ART, and the occurrence of acute events will be derived from adolescent-specific literature when available and otherwise will be derived from adult literature and varied in sensitivity analyses, as in previous work [29]. When specific interventions and studies are identified as candidates for model-based analyses, the Modeling Core will work with study teams to collect the data necessary for future model-based analyses in real time (eg, time and motion studies, activity logs, and costs of personnel and supplies).

#### Model Validation and Approach to Uncertainty

The model will be internally validated, assessing the accuracy of the model structure by comparing model output (opportunistic infections, viral suppression probability, and survival) with the empiric data in the studies from which model input parameters were derived [69]. As the model projects outcomes over lifetime horizons, the longer-term model results cannot be compared with empiric data; however, as ATN-studied interventions become more widely implemented over time, past model results will be compared with newly available data. The model will next be calibrated to data from the literature and the studies in Objectives 1 and 2 to reflect current populations of YLWH and treatment strategies. One-way, multiway, and probabilistic sensitivity analyses will be conducted, following international guidelines to address uncertainty in data inputs for the model [70,71]. This involves varying single and multiple parameters over wide ranges and reassessing all clinical results and cost-effectiveness outcomes. Sensitivity analyses can inform the potential impact of strategies in scenarios that more closely resemble programmatic rather than trial settings.

### Design for Objective 3

The design for Objective 3 was to use the computer simulation model to project the clinical impact, costs, and cost-effectiveness of interventions evaluated in ATN.

Clinical data not specific to ATN interventions will be from Objective 1, reflecting key components of disease progression and treatment for youth with and without VL suppression. Intervention-specific data will be derived from the ATN studies selected for model-based analyses; for ATN interventions, effectiveness, duration of effect, and intervention cost will be parameterized based on data from each modeled ATN trial. Model outcomes will include short-term survival and costs (calibrated to trial results) as well as projected long-term survival and costs, including life expectancy and lifetime per-person costs, and transmissions averted. To compare interventions, incremental cost-effectiveness ratios (difference in lifetime costs divided by the difference in life expectancy, in dollars per year-of-life saved) will be calculated and compared with commonly used thresholds for the United States [72]. Adolescents comprise only a small fraction of participants in HIV-specific health-related quality of life studies, and emerging data suggest that youth may attach different values to specific health states compared with adults [73-77]. Moreover, one study found that, in general, adults place less weight on impairments in mental health (eg, being worried, sad, or annoyed) and more weight on moderate to severe levels of pain, relative to adolescents [75]. In general, values attached to identical health states are typically lower for younger people in comparison with adults of all ages and may depend on the elicitation method utilized [74]. Where available, adolescent-specific utility weights will be incorporated, and the impact of utility weights on policy conclusions will be examined in sensitivity analyses, as in previous work [29].

Our work in Objective 3 will have the following 4 key areas of emphasis:

1. Work with trial teams to develop study protocols, ensuring collection of data needed for modeling.
2. Conduct pretrial modeling analyses to inform study design and establish benchmarks for interpretation of trial results. For example, to inform study design, detailed simulations of disease progression and clinical events in youth can provide additional input into sample size calculations, and model-based projections can inform study protocol elements such as frequency of study visits and maximal permitted turnaround time for return of diagnostic test results. To establish benchmarks for interpretation, model-based projections under a range of assumptions about efficacy and cost can be used to identify critical thresholds for key study outcomes: how effective and durable would an intervention need to be for that intervention to add benefit to current practice? For any given efficacy and effect duration, at what cost and level of uptake would the intervention be cost-effective?
3. Conduct modeling analyses alongside trials to evaluate the potential clinical impact and cost-effectiveness of trial interventions when implemented at scale for YLWH in the United States.
4. Identify key parameters that may influence policy conclusions such as the cost of electronic dose monitoring bottles or duration of improved adherence after incentives. If data on these key parameters are lacking, this limitation can help identify new research priorities. The Modeling Core will work with ATN leadership to ensure this feedback informs the ATN strategic planning.

### Role of the Funding Sources

The content of this manuscript is solely the responsibility of the authors and does not necessarily represent the official views of the National Institutes of Health.

## Results

This project was funded as of September 2017.

## Discussion

The planned data analyses and model development in Objectives 1 and 2 will position the ATN Modeling Core to evaluate a wide range of new ATN studies and other emerging data. Future work may include new therapies that are likely to be studied in the near future among YLWH, for example, long-acting ART [78]. The Modeling Core also collaborates with the ATN Data Harmonization Working Group to standardize the collection of resource utilization and cost data across all active ATN studies [79].

### Strengths and Limitations

This protocol has several limitations inherent to model-based analyses. First, many models necessarily use short-term data to project across longer-term horizons. This extrapolation requires assumptions about whether and how trial-derived clinical risks and costs will change over time. However, when these assumptions are clearly described, examined rigorously in sensitivity analyses, and interpreted appropriately, this ability of models to leverage short-term data into longer-term policy recommendations is one of the key strengths of model-based approaches [4,69]. Second, research participants in Objective 1 studies may not be representative of the larger population of YLWH in the United States. However, these studies remain among the best sources of data for YLWH in the United States. Study-derived risks will be varied widely in model-based sensitivity analyses to examine the potential impact of variations in these results.

### Conclusions

In summary, a Modeling Core has been established within ATN 161. A computer simulation model reflecting disease progression, care and treatment outcomes, and HIV transmission among adolescents and young adults will be developed. YLWH are a growing and vulnerable population in the United States, in whom lack of VL suppression contributes to poor clinical outcomes for individual patients, increases health care costs, and drives the ongoing HIV epidemic. Existing data from completed and ongoing NIH-supported studies will be leveraged to develop the adolescent-specific model. The Modeling Core Steering Committee will work closely with ATN leadership and investigators to design and conduct model-based analyses, addressing critical questions about HIV care among YLWH that cannot be fully answered by trials and cohort studies. The Modeling Core will also build a foundation to inform the design of new studies of interventions across ATN and to evaluate the clinical impact and cost-effectiveness of those interventions, directly translating the work of ATN into critical policy recommendations for YLWH in the United States.

## Abbreviations

ART: antiretroviral therapy
ARV: antiretroviral
ATN: Adolescent Medicine Trials Network for HIV/AIDS Interventions
CDC: Centers for Disease Control and Prevention
CD4: cluster of differentiation 4
CEPAC: Cost-Effectiveness of Preventing AIDS Complications
IMPAACT: International Maternal, Pediatric, and Adolescent AIDS Clinical Trial
NIH: National Institutes of Health
NPHIVY: nonperinatally HIV-infected youth
PHIVY: perinatally HIV-infected youth
VL: viral load
WHO: World Health Organization
YLWH: youth living with HIV
